# Hyperphosphatemia in patients with ESRD: assessing the current evidence linking outcomes with treatment adherence

**DOI:** 10.1186/1471-2369-14-153

**Published:** 2013-07-18

**Authors:** Adrian Covic, Anjay Rastogi

**Affiliations:** 1 “Gr.T. Popa" University of Medicine and Pharmacy, Iasi, Romania; 2 C.I. Parhon Hospital, Blvd Carol 1st Nr 50, Iasi 6600, Romania; 3 University of California, Los Angeles, CA, USA

**Keywords:** Adherence, Chronic kidney disease, Hyperphosphatemia, Phosphate binder, Pill burden

## Abstract

In recent years, the imbalance in phosphate homeostasis in patients with end-stage renal disease (ESRD) has been the subject of much research. It appears that, while hyperphosphatemia may be a tangible indicator of deteriorating kidney function, lack of phosphate homeostasis may also be associated with the increased risk of cardiovascular events and mortality that has become a hallmark of ESRD. The need to maintain phosphorus concentrations within a recommended range is reflected in evidence-based guidelines. However, these do not reflect serum phosphorus concentrations achieved by most patients in clinical practice. Given this discrepancy, it is important to consider ways in which dietary restriction of phosphorus intake and, in particular, use of phosphate binders in patients with ESRD can be made more effective. Poor adherence is common in patients with ESRD and has been associated with inadequate control of serum phosphorus concentrations. Studies indicate that, among other factors, major reasons for poor adherence to phosphate binder therapy include high pill burden and patients’ lack of understanding of their condition and its treatment. This review examines available evidence, seeking to understand fully the reasons underlying poor adherence in patients with ESRD and consider possible strategies for improving adherence in clinical practice.

## Review

### Hyperphosphatemia and outcomes in ESRD

Among patients with end-stage renal disease (ESRD), progressive deterioration of kidney function results in elevated phosphorus concentrations in tissue and in serum [1]. This has been associated with a number of clinical complications. In particular, the link between dysregulated phosphate homeostasis and increased mortality and morbidity has been demonstrated in a number of studies. For example, assessment of data from 40,538 hemodialysis patients in adjusted models revealed a stepwise increase in the relative risk of mortality associated with increasing serum phosphorus concentrations above 5 mg/dL [2]. In the retrospective United States (US) Renal Data System Waves 1, 3 and 4 study, which included 14,829 patients on hemodialysis, an association between phosphorus concentrations and the primary outcome of first cardiovascular (CV) events was observed from phosphorus concentrations of 4.5–5.3 mg/dL. Phosphorus concentrations of above 6.4 mg/dL were associated with an increased risk of all-cause mortality [3]. In addition, prospectively collected data from a 2-year historic cohort of 58,058 patients on maintenance hemodialysis showed that hyperphosphatemia (over 6 mg/dL) was consistently associated with increased risk of death [4].

These studies are among 27 studies that examined the relationship between dysregulated mineral metabolism and all-cause or CV mortality or CV events in patients with chronic kidney disease (CKD) or ESRD that were included in a systematic review. Although the authors noted limitations in the analysis owing to the low number of studies included and the quality of the data obtained from them, a greater risk of all-cause mortality was seen with elevated phosphorus concentrations: all but one of the 17 studies assessing these factors reported a significant relationship between them. Similarly, a significantly higher risk of CV mortality was reported with high phosphorus concentrations [5].

Other studies that were published too late to be included in the systematic review support its conclusions. These included the Dialysis Outcomes and Practice Patterns Study (DOPPS), a prospective cohort study in 25,588 patients with end-stage kidney disease on hemodialysis, reported an increased risk of CV mortality with serum phosphorus concentrations of 5.1–5.5 mg/dL, and an increase in all-cause mortality at serum phosphorus concentrations over 6.0 mg/dL [6]. In another prospective cohort of 10,044 patients who were beginning hemodialysis, serum phosphorus concentrations of greater than 5.5 mg/dL were associated with an increased risk of death compared with phosphorus concentrations of 3.5–4.5 mg/dL [7]. Results from a European study including 7,970 hemodialysis patients showed that those whose serum phosphorus concentrations were either above or below the National Kidney Foundation Disease Outcomes Quality Initiative (KDOQI)-recommended range had a significantly increased risk of mortality [8].

Establishing a threshold level of serum phosphorus load at which the risk of all-cause or CV mortality increases significantly will be critical for optimizing care strategies [5]. The need for control of serum phosphorus concentrations is reflected in the evidence-based Kidney Disease: Improving Global Outcomes (KDIGO) and KDOQI guidelines. These recommend the use of phosphate binders if serum phosphorus concentrations exceed those within the guideline ranges (toward the normal range for patients with Stage 5D CKD and 3.5–5.5 mg/dL for patients with Stage 5 CKD, respectively) [9,10]. However, it should be noted that no phosphate binder has been approved by the US Food and Drug Administration (FDA) for patients not on dialysis.

Despite publication of these guidelines, there is a gap between the recommendations and serum phosphorus concentrations achieved by the majority of patients in clinical practice [11]. For example, only 44% of patients in DOPPS II achieved serum phosphorus concentrations within the recommended range [12]. Precise reasons for this gap remain unclear and likely contributing factors will be examined further in this review.

### Seeking to control hyperphosphatemia and improve outcomes: use of phosphate binders

Treatment of the majority of patients with ESRD often necessitates a multimodal approach, comprising dialysis, dietary restriction of phosphorus intake and use of phosphate binders.

Dietary restriction of phosphorus intake has an important role in the management of serum phosphorus load, as evidenced by the finding that higher levels of dietary phosphorus intake and higher dietary phosphorus to protein ratios correlated with greater 5-year mortality in hemodialysis patients [13]. Educating patients to avoid major sources of natural phosphorus (often protein-rich foods) and phosphorus-containing additives (e.g., those found in soft drinks and processed meat), and to prepare phosphorus-rich foods by boiling to reduce phosphorus content, contributes to their serum phosphorus control [14]. Absorption of phosphorus via the gastrointestinal tract varies with food source, being lower for plant-based materials that include phytate and higher for foods enhanced with inorganic phosphorus-containing preservatives [14].

However, the balance between maintaining sufficient protein intake and restricting phosphorus intake, and effect on clinical outcomes, can be complex. It is recommended that dietary protein intake in clinically stable dialysis patients should be at least 1.1 g protein/kg ideal body weight/day [15]. However, higher protein intake (up to 1.4 g/kg/day, based on normalized protein nitrogen appearance) has been associated with increased survival in one study, despite an associated increase in serum phosphorus [16]. An additional consideration is that dialysis patients may be more prone to malnutrition resulting from a low-protein diet and/or lack of appetite [17]. Indeed, one *post hoc* analysis carried out on data from 1,751 hemodialysis patients found that prescribed dietary phosphorus restriction was not associated with survival benefit [18]. As such, there has been much debate about the potential benefits and risks of limiting dietary phosphorus intake in dialysis patients [16,17]. Furthermore, dietary phosphorus restriction may be associated with longer-term impracticalities and is often insufficient to maintain phosphate control [19]. This problem is exacerbated by the fact that, in addition to foods naturally rich in phosphate, phosphate additives are commonly found in processed foods that may not always be labeled accordingly but contain a high, often ‘hidden’, phosphate load [20,21]. This makes restriction of phosphate intake increasingly challenging for patients.

A typical phosphorus-restricted diet for hemodialysis patients includes ~900 mg of phosphorus per day, of which ~371 mg are absorbed [22]. Therefore, additional means of reducing serum phosphorus load are necessitated, and most patients require the use of phosphate binders [23]. Phosphate binders can provide the patient with greater nutritional freedom. However, different types of binders have different phosphate binding capacities; for example, in one study, lanthanum carbonate bound 135 mg phosphate per tablet, whereas sevelamer carbonate bound 21 mg phosphate per tablet [24]. Different phosphate binders are, therefore, associated with varying pill burden (Table 1) [25-31]. A potential means of reducing pill burden would be if patients were to adjust their phosphate binder intake according to the phosphorus content of each meal or snack [14]. It is important to note that the benefits of phosphate binder treatment should ideally extend beyond biochemical or laboratory parameters (such as serum phosphorus concentrations) to hard clinical outcomes such as CV events or prolonged survival. There are few placebo-controlled interventional studies examining the effect of phosphate binder treatment on survival. However, data from observational studies have accumulated that support the view that early treatment with phosphate binders is associated with prolonged survival [32-34]. For example, a prospective cohort study compared the 1-year mortality rate of patients initiating outpatient hemodialysis who were treated with phosphate binders during the first 90 days (n = 3,555) with that of patients who did not receive treatment with phosphate binders (n = 5,055). Patients treated with phosphate binders had a significantly lower mortality rate compared with untreated patients (multivariable-adjusted hazard ratio [HR] in the intention-to-treat analysis: 0.70; 95% confidence interval [CI]: 0.62–0.79; p < 0.0001) [32]. Treatment with phosphate binders was independently associated with decreased mortality compared with no treatment in the intention-to-treat and the as-treated analyses, with the magnitude of benefit ranging from 18–30% [32]. In another study including 23,898 patients on maintenance hemodialysis, those who were prescribed phosphate binders (n = 21,061) exhibited 25% lower mortality (HR: 0.75; 95% CI: 0.68–0.83) in models adjusted for covariates including serum phosphorus, but not adjusted for nutritional factors. Adjusting for nutritional factors somewhat reduced the strength of the association between phosphate binder treatment and lower mortality (HR: 0.88; 95% CI: 0.80–0.97). Of note, in this study, patients treated with phosphate binders had a lower mortality rate, but this may have been partly explained by better nutritional status in these patients compared with those who did not receive phosphate binder treatment [34].

**Table 1:** **Overview of currently available phosphate binders**[27,28,30,31,35-37]

**Phosphate binder**	**Mechanism of action**	Typical daily pillburden*	**Advantages**	**Disadvantages**
Aluminum salts	Aluminum binds to phosphates and forms insoluble precipitate in GI tract; aluminum hydroxide also forms compounds with phosphate ions in the blood	No safe dose identified	Effective, inexpensive	Associated with cognitive disturbances, osteomalacia and anemia. Patient requires careful monitoring
Calcium acetate (e.g., Phosex®)	Dissociation in GI tract; calcium binds to phosphates and forms insoluble precipitate	4–6 pills (1000 mg each,equivalent to 250 mgcalcium) per day	Effective and inexpensive	Potential for increased hypercalcemia; could lead to vascular calcification; high pill burden
Calcium carbonate (e.g., Calcichew)	Dissociation in GI tract; calcium binds to phosphates and forms insoluble precipitate	Pill number as prescribedper day (1250 mg each,equivalent to 500 mg calcium)	Effective and inexpensive	Potential for increased hypercalcemia; could lead to vascular calcification; high pill burden
Calcium acetate/magnesium carbonate	Dissociation of the active compounds calcium acetate and magnesium carbonate in the GI tract; each binds to phosphate and forms insoluble precipitate	Total: 3–10 pills per day(each pill contains435 mg calcium acetate/235 mg magnesiumcarbonate)	Lower calcium uptake versus calcium-based binders; effective; moderate costs	Monitoring of magnesium level required; in some circumstances, moderate increase in serum magnesium level
Sevelamer HCl	Anion exchange resin that exchanges chloride ions for phosphate ions	3 pills (800 mg each) three times daily(Total: 9 pills/day)	Effective; lipid-lowering effect; potential cardioprotective effect	Expensive; high pill burden; associated with GI side effects such as abdominal bloating, diarrhea and constipation. Potential development of metabolic acidosis
Sevelamer carbonate	Anion exchange resin that exchanges chloride ions for phosphate ions	3 pills (800 mg each) three times daily(Total: 9 pills/day)	Effective; lipid-lowering effect; potential cardioprotective effect; available as a powder, which may reduce pill burden	Expensive; high pill burden; associated with GI side effects
Lanthanum carbonate	Dissociation in the upper GI tract; lanthanum then binds to phosphates and forms insoluble, non-absorbable lanthanum phosphate complexes	1 pill (500 mg, 750 mg or 1000 mg) three times daily (Total: 3 pills/day)	Effective, low pill burden	Expensive; associated with GI side effects such as nausea, vomiting

GI = gastrointestinal, HCl = hydrochloride.

*Timing and dose of phosphate binder to be adjusted in line with timing of meals/snacks and the phosphorus content thereof.

Overall, there is a lack of long-term data from randomized, controlled studies evaluating the relative efficacy of specific phosphate binders on hard clinical outcomes such as mortality and musculoskeletal morbidity [29,38]. Furthermore, available data have been somewhat contradictory. Results of the randomized, open-label Dialysis Clinical Outcomes Revisited study in 2,103 hemodialysis patients showed no difference in the primary endpoint of all-cause mortality among patients receiving sevelamer hydrochloride (HCl) compared with those receiving a calcium-based phosphate binder, although a significantly lower risk of mortality was reported in patients ≥65 years receiving sevelamer compared with those receiving a calcium-based binder (p = 0.02) [39]. These data contrast with results from the randomized, open-label Renagel In New Dialysis study in 148 patients new to hemodialysis who were randomized to receive calcium-based phosphate binders or sevelamer HCl. In this study, the 18-month coronary artery calcification score increased significantly in each treatment group but was significantly larger with calcium-based binders than with sevelamer (p = 0.01) [40]. After a median follow-up of 44 months, mortality was significantly higher in patients receiving calcium-based binders than in those receiving sevelamer (p = 0.05) [41]. In line with these observations, the prospective, randomized Treat To Goal study in 200 hemodialysis patients reported no significant differences in serum phosphorus and calcium × phosphorus product between patients receiving calcium-based phosphate binders and those receiving sevelamer HCl from baseline to 52 weeks. However, patients receiving a calcium-based binder showed significant progression in coronary artery and aortic calcification at Week 52, whereas those receiving sevelamer HCl showed no significant change in these parameters [42]. Results such as these have prompted concerns that the combined long-term effects of dysregulated mineral metabolism and its treatment with calcium-based phosphate binders may contribute to calcium overload and vascular calcification [43]. Results from a recently published pilot clinical trial in 148 patients with moderate CKD and near-to-normal serum phosphorus concentrations have not clarified this matter. These data reiterated the efficacy of phosphate binders in reducing serum phosphorus concentrations, but showed progression of arterial calcification with phosphate binder treatment in these patients compared with placebo. The apparent adverse effects on vascular calcification were more pronounced among patients randomized to calcium acetate, although patient numbers were low in this subset analysis [44]. It should be emphasized that the patient population selected for this study was different to those in previous studies in this area. Until these datasets can be fully explained, the role of the phosphate binders – and specifically different types of phosphate binders – in changing outcomes, in patients with CKD who have not yet progressed to ESRD and in patients with ESRD, will remain a focus of future research.

Currently available phosphate binders differ in their overall effectiveness in the short and long term, their mode of action, composition and tolerability [22]. A number of factors may contribute to the differences in effectiveness between currently available phosphate binders, one of which is poor adherence.

### Lack of treatment adherence: a problem of chronic illnesses or specific to ESRD?

Poor adherence to prescribed drug therapy is a well-established obstacle to treatment success, with efforts to improve it hampered further by a lack of consistency in defining and measuring adherence across existing studies [45]. One meta-analysis of 21 studies including 46,847 patients with various conditions such as recent myocardial infarction, HIV and type 2 diabetes showed that patients with good adherence to drug therapy had a significantly reduced risk of mortality compared with patients whose adherence was poor (pooled odds ratio: 0.56; 95% CI: 0.50–0.63). Interestingly, this effect extended to those who were adherent to placebo [46].

It appears that poor adherence is common in ESRD, and its detrimental impact on patients has been noted: missing hemodialysis treatments has been associated with an increased risk of mortality [47] and poor adherence to phosphate binder therapy has been associated with failure to adequately control serum phosphorus concentrations [48]. In one study, treatment adherence to phosphate binders was assessed using questionnaires in 165 hemodialysis patients. Of these, 40% were found to be non-adherent to their overall medication regimen and 21% specified that they were non-compliant with their phosphate binder regimen. Patients who did not take their phosphate binders were significantly more likely to exhibit mean serum phosphorus concentrations >5.5 mg/dL (X^2^ = 4.7; 95% CI: 1.07–6.5; p = 0.03) [48]. Overall, reported rates of non-adherence to phosphate binders vary widely across studies, from 21% to 74% [48-50]. This wide range could be attributed to differences in the definition of non-adherence between studies [45]. In addition, methodologies may differ between studies, contributing to variation in the data. For example, direct monitoring methods include drug concentration assays, use of pill markers and direct observation of pill taking; indirect methods include patient self-reports, compliance ratings by nurses, prescription refills, pill counts and microelectronic monitoring devices. All have been used to assess adherence in hemodialysis patients [45].

It has been suggested that the efficacy demonstrated by phosphate binders in clinical trials, considered together with the fact that many patients using phosphate binders have serum phosphorus concentrations outside the KDOQI-recommended range, indicate that the effectiveness of current phosphate binders is compromised by poor adherence [25]. Indeed, it has been noted that patient expectations, preference and adherence are at least as important as the efficacy of the prescribed phosphate binder [27]. In general, adherence levels are lowest in patients with chronic disease who perceive no immediate symptoms or risk if they do not take their medication, and are required to change their lifestyle in order to adhere to their prescribed treatment program [48]. Patients on dialysis fall into this category [48]. However, relatively few data are available to help us to understand other, more specific, reasons why hemodialysis patients show poor adherence to phosphate binder therapy. Available studies indicate that patients’ lack of understanding of their condition and treatment plays a role [11]. Other factors may include the cost of treatment and the possibility that physicians may prescribe increasingly high doses of phosphate binders to improve poor efficacy that may in fact be due to poor adherence. Lastly, a major reason for poor adherence is often cited as the high pill burden associated with currently available phosphate binders [49]. These are discussed in turn below.

### Treatment adherence and phosphate binders: unraveling the effect of pill burden

Dialysis patients are required to take a large number of medications. These are needed not only to control hyperphosphatemia, but also to manage a number of other conditions such as diabetes or hypertension. The importance of reducing pill burden and simplifying regimens to improve adherence has been highlighted in other diseases, such as HIV [51] and CV disease [52]. In ESRD, the complexity of combined treatment regimens presents a challenge for both prescribers and patients [45]. A study assessing the medication prescribing patterns for 10,474 ambulatory hemodialysis patients in the Dialysis Clinic, Inc. database revealed that the number of medications being prescribed is increasing, with a mean of 12.3 ± 5 different medications being used by each patient [53]. Of these patients, 88% were using phosphate binders [53].

Results from a study of 233 patients on maintenance dialysis from three different units in the US showed that patients took a mean of 11 ± 4 medications (nine oral, two parenteral), with a median daily pill intake of 19 (inter-quartile range [IQR]: 12) [49]. Phosphate binders accounted for 49 ± 19% of the total pill burden, with a median pill count of 9 (IQR: 6) [49]. Only 38% of patients in this study were adherent to their prescribed phosphate binder therapy (taking 80–120% of expected pill count), and adherence decreased significantly with increased pill count (r = −0.19; p = 0.006). Importantly, median serum phosphorus concentrations were 5.2 mg/dL (IQR: 1.4) [49]. Furthermore, multivariate analyses showed that a high total pill burden was independently associated with low physical component summary scores of the short-form (SF)-36 quality of life assessment, although no relationship was observed between the mental component of SF-36 and pill burden [49].

In summary, a higher phosphate binder pill burden has been associated with lower adherence and higher serum phosphorus concentrations [48,49]. Further investigation to establish the relative impact of frequency of administration and reduction in pill number taken at each administration would be of interest and could further guide clinical decisions. Furthermore, alternative formulations of phosphate binders, such as powder formulations, have the potential to reduce pill burden and further support adherence [54,55]. In the meantime, efforts to reduce the pill burden associated with phosphate binders should continue in an effort to improve adherence.

### Treatment adherence is multifactorial

As with treatment of many chronic diseases, patient socioeconomic status, education and demographics can affect adherence. An important factor is patient age. Although it has been reported that older patients (>65 years) tend to be more adherent to treatment [45], the high prevalence of cognitive impairment among the hemodialysis population, in particular during dialysis itself (when many conversations about medication take place), may lead to confusion about complex medication regimens [56].

In a study of 188 dialysis patients, 129 of which were receiving hemodialysis, the primary reason given by patients for their non-adherence was being unaware of the correct prescription (37%). This was followed by patients forgetting their pills (30%). Interestingly, patients who admitted to poor adherence were prescribed more phosphate binders, and took fewer of them, than their adherent counterparts [57]. This indicates that as patients do not always report their non-adherence, their physicians may increase the prescribed dose in an attempt to re-establish phosphate control, initiating a cycle of increases in pill burden and attendant reduction in adherence. In support of this, another study reported that patients who admit to being non-adherent and exhibit higher phosphorus concentrations are being prescribed the highest doses of binders [48].

A further possibility is that patients’ lack of understanding of hyperphosphatemia, its management and its potentially detrimental impact on their health may affect adherence [45]. Hyperphosphatemia, like many other chronic diseases including hypertension, dyslipidemia and osteoporosis, is a silent condition until the manifestation of potentially fatal symptoms. This disconnect between the potential severity of the condition and patients’ understanding of its impact on their health could be addressed with tailored education initiatives.

Lastly, existing phosphate binders are associated with different adverse effect profiles (Table 1), which should be considered in tandem with their clinical efficacy and, together with pill burden, may affect phosphate binder choice or patient satisfaction. This highlights the importance of offering a wider choice of medication, enabling physicians to customize treatment to the needs of individual patients.

### How can we improve adherence in patients with ESRD?

Interventions to improve treatment adherence in the long term often comprise multiple components including improved education, reminders, self-monitoring, telephone follow-up and supportive care. Despite this, approaches in chronic disease have often had limited effect [58]. For patients with ESRD, a number of strategies could be investigated (or investigated further; Table 2). Of these, patient education is likely to play an important role in improving adherence in ESRD, and, with it, control of serum phosphorus concentrations. Some examples of successful patient education programs have been published. For example, in a randomized, parallel-group, controlled study including 56 hemodialysis patients, those who attended an education session about phosphate management showed a significant reduction in serum phosphorus concentrations after attending. This was maintained up to 3 months, whereas no significant change in serum phosphorus concentrations was observed in the control group [59]. Similarly, 34 patients with stage 4–5 CKD, most of whom were on hemodialysis, who attended a 2-month structured educational program as part of a smaller, non-randomized study exhibited significantly reduced plasma phosphate up to 12 months [60]. Finally, in a study of a nurse-led intervention, 41 hemodialysis patients attended an education session about phosphate binders 5 weeks into the study period, followed by bi-weekly personalized counseling sessions until Week 17 (end of study). Adherence to phosphate binders increased from 83% to 94% over the study period, compared with a reduction from 86% to 76% in historic controls. This was matched by a reduction in serum phosphorus concentrations from 4.9 to 4.3 mg/dL in patients on the study [61]. Again, lessons can be learned from the long-term treatment of other conditions: a pharmacy care program including education initiatives and custom-packaged medication led to a significant and sustained improvement in adherence after 14 months in patients taking at least four daily medications for chronic conditions [62]. Initiatives such as this may require greater coordination of care and sharing of patient data between different care settings [63].

**Table 2:** Potential strategies to improve control ofdietary phosphorus intake and adherence to phosphate binders in patients with ESRD

**Patient education**	• Introduce education programs, led by nurses or other ancillary healthcare providers,focusing on the:
	◦ Physiologic role of phosphate and its presence in different foods
	◦ Role of phosphate in ESRD-associated cardiovascular disease
	◦ Importance of phosphate binders and their role in lowering serum phosphorusconcentrations
	◦ Importance of dietary adherence
	• Involve patients’ families and friends in education initiatives
	• Tailor education to patient’s lifestyle, environment, career, ethnicity, cultural background and socioeconomic status
	• Educate patients on appropriate food choices and provide training on preparing suitable meals
**Patient empowerment**	• Introduce initiatives such as the ‘Phosphate Education Program’ that enable patients with hyperphosphatemia to estimate the phosphate content of their meals and adjust their phosphate binder dose accordingly
**Improve properties of phosphate binders**	• Reduce pill size and burden
	• Improve palatability
	• Reduce associated adverse effects
	• Introduce electronic monitoring devices, which may help patients to remember to take their medication and support adherence

The success of patient education initiatives relies to some extent on patients themselves being fully engaged with their treatment. Patients have to contend with the fact that dietary phosphorus content has been shown to vary substantially depending on the type of meal [64], with the subsequent potential for inappropriate, particularly under-, dosing [64]. Empowered patients can apply knowledge about their treatment to adopt a suitably flexible approach, for example to counteract potential shortcomings in a typical fixed-dose phosphate binder regimen by adapting the timing and level of their phosphate binder intake on a meal-by-meal basis [64]. A prospective study which may serve as an example of this approach was based on patient empowerment and family support. Parents of children with various stages of CKD (pre-dialysis and on dialysis) estimated the inorganic phosphorus content of meals and adjusted phosphate binder dosage accordingly at each meal time. Mean serum phosphorus concentrations decreased significantly up to 12 weeks after the initial workshop. The study also showed a relatively high rate of patient adherence to the program (median 8.0 on a scale of 1–10), increased phosphate binder consumption and increased patient satisfaction at meal times [65]. The approach used in this study was the Phosphate Education Program (PEP), which applies the concept of a ‘Phosphorus Unit’ to indicate the phosphorus content of food groups, allowing patients to quickly estimate the phosphorus content of their meals without referring to complex tables or calculations, and to adjust their phosphate binder intake accordingly [64]. As such, the PEP represents a novel approach, integrating patient empowerment into the management of hyperphosphatemia [64].

Simplification of dosing regimens by reducing pill burden and size, and improving the palatability of phosphate binders is also likely to improve patient satisfaction and, with it, adherence. For example, calcium acetate gelcaps are easier to swallow than tablets and are reportedly preferred by patients [66]. Patient and physician preference data have also been reported in one study that compared lanthanum carbonate with patients’ previous phosphate binders. One of the principal reasons for patients’ stated preference for lanthanum carbonate was ‘number of tablets’ [67].

Currently, few randomized, controlled studies provide an insight into differences in adherence between phosphate binders. Given the considerations above, it has been stated that an ideal phosphate binder should be associated with few adverse effects; have high efficacy regardless of pH, good palatability and no or minimal absorption in the digestive tract; require low daily dosing; and be available at a low cost [68]. Until this ideal is met, treatment tailored to the individual patient and their circumstances is required.

## Conclusions

In order for us to update guidelines and optimize patient care, some gaps in the evidence base need to be addressed. Although available data demonstrate the association between hyperphosphatemia and increased risk of mortality, it remains a challenge in the clinic to achieve and maintain recommended serum phosphorus concentrations. A major obstacle to doing so is the problem of patient adherence to diet and phosphate binders. We suggest that an approach to improve patient adherence should comprise three components: firstly, standardization of methods for measuring and monitoring adherence between studies in ESRD; second, scientific validation of strategies to improve adherence, such as patient reminders and comprehensive patient education; and lastly, continued clinical research into development of new formulations and/or phosphate binders with a reduced pill burden.

## Competing interests

AC has received lecture and consulting fees from Amgen, Abbott, Fresenius, and Vifor. AR has taken part in speaker bureaus for ViiV Healthcare Systems, Sanofi/Genzyme, Questcor and Cubist and has taken part in advisory boards for Vifor.

## Authors’ contributions

AC and AR both participated in deciding the overall concept of the manuscript, drafting the manuscript and reviewing the manuscript at each stage of its development. Both authors read and approved the final manuscript.

## Pre-publication history

The pre-publication history for this paper can be accessed here:

http://www.biomedcentral.com/1471-2369/14/153/prepub
